# Cannabis use patterns, motivations, and reasons for abstinence in pregnancy

**DOI:** 10.3389/fpsyt.2025.1613324

**Published:** 2025-10-23

**Authors:** Lisa M. Blair, Meghna Shukla, Julie A. M. J. Kurzer, Marvin Schilt-Solberg, Biyyiah A. Strickland, Salma Akter, Dennette Fend, Kimberly Hamann, Kristin Ashford

**Affiliations:** ^1^ College of Nursing, Wayne State University, Detroit, MI, United States; ^2^ School of Nursing, University of Michigan, Ann Arbor, MI, United States; ^3^ Nursing, University of Kentucky, Lexington, KY, United States

**Keywords:** pregnancy, prenatal, cannabis, tobacco, motivations

## Abstract

**Introduction:**

Prenatal use of cannabis, and co-use of tobacco, has escalated rapidly despite well-documented risks to pregnancies and offspring. The purpose of the present study was to examine relationships between prenatal cannabis use, motivations for use, reasons for abstinence, co-use with tobacco and nicotine, and quit attempts among a cohort of persons who used cannabis in their current pregnancy.

**Methods:**

Persons who used cannabis at least once during their current pregnancy were recruited from prenatal clinics and surveyed. Descriptive statistics and logistic regression (*n* = 59) were used to determine differences between those who had continued (past 30-day) use of cannabis compared to those who did not.

**Results:**

The data reveal that motivations for use and reasons for abstinence of cannabis are complex, with many participants indicating past, unsuccessful attempts to quit. Current cannabis use (past 30 days) was reported by 61% of participants, with 54% of those endorsing daily use and 85% endorsing use at least 15 of the past 30 days. Those who endorsed five or more motivations for use were over 10 times as likely to have recent cannabis use.

**Discussion:**

This study highlights major research gaps and discusses clinical and policy implications of the findings and of perinatal cannabis.

## Introduction

Recent epidemiological data suggests more than 1 in 8 U. S. pregnancies is affected by prenatal cannabis use, a rapid escalation in use over nearly two decades of trend data (1, 2). Similar escalation in use has been seen in other facets of the U. S. population, concurrent to legalization efforts and the rapidly emerging science surrounding the medicinal use of cannabis and cannabinoids (3). Yet evidence about cannabis use in pregnancy remains underdeveloped, due in large part to ethical concerns about exposure of pregnancies to a substance with well-established associations with maternal and fetal harms. Furthermore, recent changes to the potency of available cannabis products, five-fold more powerful today than twenty years ago (4), and legal context around use and possession of cannabis creates significant challenges when attempting to generalize older literature to modern contexts.

Recent, well-conducted meta-analyses and large-scale studies have established that prenatal cannabis exposure has a dose-dependent relationship with low birth weight (5, 6) and is associated with heightened risks of preterm birth (7); stillbirth (1); Autism Spectrum Disorder (8); Attention Deficit Hyperactivity Disorder (9); and maternal hypertension, preeclampsia, and placental abruption (10). Recent work has also established that timing of *in utero* exposure may be critical, with first-trimester exposures and exposures across pregnancy resulting in more severe effects on birth weight and head circumference compared to other timings (6). Though these studies do not establish a causal link, the consistent and concerning evidence of harm, coupled with strong biological plausibility, offer increasingly robust support for recommendations to avoid cannabis use in pregnancy. National professional bodies, including the American College of Obstetricians and Gynecologists (ACOG) and the American Academy of Pediatrics (AAP) recommend that cannabis use be discontinued or avoided in persons who are pregnant or intending to become pregnant (11, 12). Yet pregnant persons report largely perceiving cannabis as safe to use (13).

Pregnant women have reported using cannabis to treat chronic pain, insomnia, nausea and vomiting, and mental health symptoms and to relax (14, 15). These unmet medical and mental health needs, coupled with enhanced perceptions of safety and acceptability, may be driving the rapid rise of cannabis use rates in pregnancy as a harm reduction strategy. This is particularly underscored by documentation of preferences for 'natural' treatment vs. traditional medical therapies in the general population of individuals who use medical cannabis (16). Thus, understanding the motivations of pregnant persons to use cannabis is critical, both toward the development of effective intervention and prevention efforts and for clinicians who are attempting to counsel patients.

Cannabis, while often perceived as less addictive and less harmful compared to other substances, carries the risk of cannabis use disorder (CUD). Studies estimate that approximately 30% of people who use cannabis will develop physical symptoms of dependence (17), with the risk increasing among those who begin during adolescence (18). CUD is characterized by impaired control over use, significant social or occupational disruption, continued use despite health risks, and physiological dependence (19). Among pregnant individuals, rates of cannabis dependence and CUD (later combined in the DSM-V) rose over 5-fold from 1993 to 2014 (20) and were notably higher than in the general population of women, underscoring the need for early identification and intervention to mitigate risks for both maternal and fetal health.

Tobacco, including combustible forms (e.g., cigarette smoking, hookah) and its metabolites (e.g., nicotine), are commonly co-used with cannabis in the general population and in pregnancy, with age of initiation in both substances serving as an important predictor of future behavior (21). Nicotine is the primary psychoactive constituent of tobacco and the refined derivative commonly used in vaporizing liquids for use in electronic nicotine delivery systems (ENDS); it is a known human teratogen and class D drug in pregnancy, with well documented adverse maternal and child health outcomes similar to those described for cannabis use above (22), making it a potent confounder to cannabis in outcomes-focused research. Indeed, concurrent use of tobacco with cannabis has been seen as nearly ubiquitous in pregnancy. Nationally representative data from the Population Assessment of Tobacco and Health (PATH) study revealed only 2.19% of pregnant women between 2014–2018 used cannabis without also engaging in tobacco use (23); this study also revealed the use of tobacco as a significant predictor of continued cannabis use during pregnancy. Modern studies of cannabis use in pregnancy attempt to control for tobacco using statistical methods to offset this challenge, and researchers including the present team have called for collection and reporting of tobacco data in all studies involving prenatal use of cannabis.

Motivation is an important concept in behavior theories and substance use research. The originator of the Theory of Planned Behavior viewed motivations as the variables which influence behavior (24). According to this theory, the greater the motivations to engage in a specified behavior, the greater the likelihood that the individual will perform the behavior (25). Similarly, the Health Belief Model views individuals as logical beings who use a rational approach to make health-related decisions (26). In this view, all individuals are believed to possess the motivation to avoid poor health (27). Motivation in this context is influenced by the individuals' perception of personal susceptibility to an unwanted health condition, the seriousness of that condition, the benefits of taking action to avoid the condition, and the barriers to taking action. These and other theories of behavior and behavior change highlight the importance of understanding motivations for substance use behaviors.

The current investigation sought to elucidate cannabis use patterns, motivations for use, reasons for abstinence, concurrent use of tobacco, and prior quit attempts in a cross-sectional cohort of pregnant persons who had used cannabis in their current pregnancy. Better understanding of these critical drivers of behavior will aid in addressing the rapid uptake of cannabis use in pregnancy.

## Materials and methods

Sixty pregnant persons (*n* = 60) were recruited for the study at four prenatal clinics affiliated with an academic medical center serving urban, rural, and Appalachian counties in central Kentucky from March to October 2023. Eligibility criteria were: 1) age 18-45, 2) currently pregnant, and 3) had used cannabis at least one time during the current pregnancy (self-report), even if it was before they knew they were pregnant. Those who met eligibility criteria and agreed to participate were given a link to complete an online survey following their prenatal care visit. Participants were provided with compensation for their time in the form of a $25 electronic gift card. A Certificate of Confidentiality, issued by the National Institutes of Health, was obtained prior to enrollment, and participants were protected by study procedures from triggering mandatory reporting requirements. The University of Kentucky Institutional Review Board approved this study via protocol number 87476-2019. This pilot study was intended to support development of a larger study examining prenatal cannabis use trends, motivations, and reasons for abstinence across pregnancy.

### Cannabis policy context of study

At the time of data collection (May 2023 - October 2024), cannabis remained a Schedule I drug at the federal level in the location where the study was conducted (United States). The legalization of cannabis for medical and recreational purposes was contemporaneously being discussed in the Kentucky legislature. In March 2023, the legislature passed into law a bill allowing limited medical use, but this did not take effect until January 2025, meaning that in Kentucky, possession of cannabis was illegal for all persons and purposes within the state during data collection for the present study (28). A governor's executive order specified a path to pardon for individuals convicted of possession for small quantities of cannabis under specific conditions that required documentation of at least one listed medical condition and legal purchase from a medical cannabis dispensary in another state (29). As of July 2025, no legislative effort has succeeded in legalizing cannabis for recreational use in the state, though multiple proposals exist, including two that would place the measure on the ballot for voters (30). Kentucky also had a rule in effect at the time of study collection classifying prenatal substance use as child abuse and mandating reporting of any infant who tested positive for illicit substances at birth. Thus, participants may have been hesitant to participate in this convenience sample. Despite these limitations, recruitment for the study was completed in a quarter of the time estimated in the study protocol. Participants reported being eager to learn more about safety and risks of cannabis use in pregnancy.

### Substance use variables

Modes of consumption (modality) of cannabis and tobacco vary across populations and may have varying health effects. Importantly, shifts in the availability and prevalence of alternate modalities (e.g., waxes, dabs, ENDS) and rising use rates beyond traditional forms of smoking cannabis or tobacco require more comprehensive measurement to paint a full picture of substance use behaviors and dependence (31). Importantly, participants may also have understandings of modalities that differ from researchers (e.g., ENDS not being understood as a form of tobacco despite including nicotine), leading to systematic survey error in the absence of comprehensive assessment of modality (32). Though characterization of modality is not a primary endpoint of the present study, descriptive statistics are reported for these variables to inform the field and allow contextual understanding of the sample and results. Participants were asked whether they had used tobacco or cannabis via any modality in the past 30-days with examples provided and options to add 'other' forms if needed. Those who responded yes were asked on how many days of the last 30 they had used the substance (range: 1-30). Past 30-day cannabis use and past 30-day tobacco use variables were then created as a composite informed by all measured modalities of use. We chose past 30-day use as a metric due to concerns of recall bias and to ensure that the measurement period captured only use that occurred during pregnancy for all participants.

Past quit attempts were assessed in the full sample for both cannabis and tobacco. Participants were asked if they had ever attempted to quit using [substance], how many times they had tried to quit, what methods they had used to support a quit attempt, and how motivated they were to quit now. Logical skip patterns (i.e., not asking how motivated someone was to quit if they had no current use) reduced sample size for some quitting questions, and this is explained in the results where appropriate.

### Motivations for use and reasons for abstinence

Motivations were assessed using a panel of 18 yes/no questions describing common and pregnancy-specific motivations for cannabis use. A composite measure was then constructed using a summative scoring method (possible range: 0-18). The composite was then dichotomized to 0–4 or 5+ motivations to examine the odds of use based on number of motivations endorsed. Reasons for abstinence from cannabis was measured using six questions with dichotomous response options (yes/no): "uncertain about safety," "illegal where I live," "cost," "concerns about effects on baby/pregnancy," "just didn't want to," and "other reason" with option to free text response. Reasons for abstinence from tobacco included five of the six questions but excluded "illegal where I live." Motivation and reasons questions were generated from review of the literature on prenatal cannabis, the prior research of the present study team, and researcher interactions with Survivors Union of the Bluegrass, a community advisory board composed of people who use drugs and/or people in non-abstinence-based recovery. Several members of the Survivors Union of the Bluegrass had used cannabis during previous pregnancies. Motivations for use and reasons for abstinence questions were not derived from either the Theory of Planned Behavior or Health Belief Model, specifically, but align with the conceptualization of motivation as a predictive driver of behavior that is common to both behavior change theories. While this measure has not been validated in a large sample to date, no validated measures exist for motivations for cannabis use in pregnancy. Extant literature relies heavily on investigator-created measures for motivations for use and/or qualitative methods of assessment.

### Covariate selection

For this analysis, we selected maternal age (coded continuously) and past 30-day tobacco use (coded dichotomously) as covariates. Age is a significant predictor of drug use in general and in pregnancy, including cannabis and tobacco (33), in extant literature and may confound likelihood of experiencing pregnancy symptoms and other factors specific to this population that participants have endorsed (i.e., reported or noted the presence of) in prior research (e.g., pain). Tobacco is the most commonly co-used substance with cannabis across populations, and the most commonly used substance with addictive potential in pregnancy (34). Historical data on usage trends suggest that tobacco use is both a predictor of cannabis use and a powerful confounder for cannabis use outcomes (21).

### Analysis

Data were visualized and examined for missingness using SPSS version 30.0. The survey was designed using skip logic to reduce participant burden. Values for frequencies affected by skip patterns were logically assigned such that if a participant triggered a skip pattern by answering that they had never used a substance, the frequency of use for that substance was coded as zero. Individuals who indicated they had never used cigarettes, hookah, or ENDS were not included in the calculation of means and standard deviations for age of initiation for substances they had not used. The analysis sample consisted of 59 individuals with valid responses to any cannabis question. Missing data on variables of interest in the analysis sample was limited to one individual who did not answer the question about maternal age and 3 who did not respond to questions about past quit attempts.

Data analysis was performed using SPSS 30.0. Descriptive statistics were calculated for all variables. Fisher's Exact chi-square (*χ^2^*) and t-tests were performed, as appropriate, to evaluate differences between those who endorsed past 30-day cannabis use and those who did not. Odds ratios (OR) of group membership (past 30-day use, no past 30-day use) and 95% confidence intervals (CI) were calculated for each of the 18 motivations but reported only for those motivations that had *p* <.05. Two logistic regression models were fit to assess the impact of multiple motivations on odds of group membership. First, maternal age and current smoking status were examined as predictors without motivation (empty model). Then the dichotomous composite measure (0–4 or 5+) was included (full model). Our primary test was of the effect of composite motivations on past 30-day cannabis use; therefore, we did not correct for multiple testing in individual indicators or descriptive statistics beyond the familywise omnibus full model logistic regression. We further conducted a *post hoc* binary logistic regression of the full model with only participants in the second and third trimester as a sensitivity analysis to determine if associations were driven by early pregnancy use patterns and/or use prior to pregnancy diagnoses.

## Results

Participants (*n* = 59) ranged from 18 to 38 years old, with a mean age of 26.48 years (SD = 4.64). Gestational age at time of survey ranged from 6 to 40 weeks, with a mean of 20.69 (SD = 11.51). All participants had received at least one prenatal care provider visit at time of survey completion. Most participants identified as White (86.4%), married or cohabitating (57.6%), having at least some college or trade school (52.6%) and with household incomes of less than $50,000 per year (60.8%). Participant characteristics in the full sample and when divided by past 30-day use of cannabis are available in **Table 1**.

**Table 1:** Demographics at time of survey completion for full sample and by past 30-day cannabis use status.

Characteristic	Full sample *n* = 59	No past 30-day use *n* = 23 (39.0%)	Past 30-day use *n =* 36 (61.0%)	p
Age (years; range 18-38)	26.48 [4.64]	25.64 [4.67]	27.00 [4.60]	.046
Weeks gestation (range 6-40)	20.69 [11.51]	24.57 [12.15]	18.22 [10.51]	.281
Race/ethnicity				
White	51 (86.4)	32	19	.126
Black or African American	3 (5.1)	0	3	
Asian	1 (1.7)	1	0	
More than one race	4 (6.8)	3	1	
Marital status				
Married or partnered	34 (57.6)	18 (50.0)	16 (72.7)	.067
Single	23 (39.0)	18 (50.0)	5 (22.7)	
Divorced or separated	1 (1.7)	0 (0.0)	1 (4.5)	
Education				
Less than high school	3 (5.1)	3 (8.3)	0 (0.0)	.685
GED	7 (11.9)	4 (11.1)	3 (13.0)	
High school diploma	18 (30.5)	11 (30.5)	7 (30.4)	
Some college or trade	25 (42.4)	15 (41.7)	10 (43.5)	
College graduate	6 (10.2)	3 (8.3)	3 (13.0)	
Employment status				
Employed part-time	11 (18.6	8 (22.2)	3 (13.0)	.119
Employed full-time	24 (40.7)	11 (30.6)	13 (56.6)	
Unemployed/seeking	10 (16.9)	6 (16.7)	4 (17.4)	
Homemaker	11 (18.6)	10 (27.8)	1 (4.3)	
Student	2 (3.4)	1 (2.8)	1 (4.3)	
Other (e.g., self-employed)	1 (1.7)	0 (0.0)	1 (4.3)	
Household income				
Less than $20,000	23 (39.0)	16 (44.4)	7 (30.4)	.171
$20,000 to $49,999	22 (37.3)	15 (41.7)	7 (30.4)	
$50,000 or more	9 (15.3)	3 (8.3)	6 (26.1)	
Don't know	5 (8.5)	2 (5.6)	3 (13.0)	

Continuous variables are reported as mean [SD] and categorical variables are reported as *n* (% of total for that column). *p values* are reported for comparison between those with past 30-day use and those without past 30-day use. GED, general education degree.

### Substance use behaviors

#### Ever use

All participants reported cannabis use, and 88.1% reported having ever used a tobacco or nicotine product (e.g., cigarettes, hookah, ENDS). Frequency of type of tobacco or nicotine varied, with 74.6% having smoked cigarettes, 23.7% having used hookah, and 62.7% having vaped nicotine. Other tobacco products reported by participants (free text) included chewing tobacco (*n* = 1), cigar (*n* = 2), cigarillo (*n* = 3), dip (*n* = 1), and snus (*n* = 1). Sixty-four percent of participants reported having smoked at least 100 cigarettes in their lifetime. While a higher raw percentage of those who endorsed recent cannabis use (past 30 days) had ever used a tobacco product (94.4%) compared to those without recent cannabis use (78.3%), the difference was not statistically significant (*p* = .061).

#### Age of initiation of use

Cigarette smoking (mean age = 15.58 years, SD = 3.32) and cannabis (mean = 15.48, SD = 2.94) had similar age at first use. Hookah (mean age = 17.00, SD = 2.41) and ENDS (mean = 18.34, SD. 4.49) were taken up slightly later. There were no statistically significant differences in age of onset of any of the tobacco products when examined across participants who endorsed recent (past 30-day) cannabis use compared to those who did not (*p*s >.05). Substance use patterns and age of initiation are presented in **Table 2**.

**Table 2:** Substance use patterns and age of initiation of substance use, by substance.

(Range, *n*, %)	Full sample mean [SD]	Range (years)	Full sample *n* (%)	No past 30-day cannabis use *n =* 23 (39.0%)	Past 30-day cannabis use *n* = 36 (61.0%)	*p*
Ever used tobacco or nicotine			52 (88.1)	18 (78.3)	34 (94.4)	.061
Age at first use (in years)						
Cannabis	15.58 [3.32]	10-26	59 (100)			
Cigarettes	15.48 [2.94]	8-25	42 (71.2)	16.40 [3.64]	14.96 [2.39]	.065
ENDS (nicotine)	18.34 [4.49]	9-29	35 (59.3)	16.64 [4.65]	19.13 [4.29]	.065
Hookah	17.00 [2.41]	13-22	12 (20.3)	16.75 [3.86]	17.13 [1.64]	.407
Past 30-day use of cannabis						
Yes			36 (61.0)			
No			23 (39.0)			
Days of cannabis use in past 30 among those who endorsed any	21.89 [10.27]					
Past 30-day use of cigarettes						<.001
Yes			22 (37.3)	2 (8.7)	20 (55.6)	
No			37 (62.7)	13 (56.5)	9 (25.0)	
Past 30-day use of ENDS						.076
Yes			21 (35.6)	4 (17.4)	17 (47.2)	
No			38 (64.4)	7 (30.4)	8 (22.2)	

ENDS, electronic nicotine delivery systems (e.g., e-cigarettes or vapes).

#### Modes of cannabis consumption

All but two participants indicated that they smoked cannabis (96.6%). Eating products that contain cannabis (e.g., candy, baked goods, gummies) was a mode of consumption for 80.0% of participants. Vaping cannabis with an e-cigarette or other device was the third most common form, used by 76.3% of participants. Other modes of use provided by participants (free text) included water pipes (e.g. dabs, rigs, waxes, *n* = 4), THC patches (*n* = 1), and 'medicinal rub' (*n* = 1).

#### Past 30-day

In the full sample, 61.0% of participants reported cannabis use in the past 30 days. Among those endorsing past 30-day use, the mean number of days cannabis was used was 21.89 days (SD = 10.27, range: 2–30 days). Most participants with past 30-day use reported using cannabis at least 15 days in the past 30 days (*n* = 30, 83.4%), with more than half reporting daily use (*n* = 20, 55.6%). Cigarette use in the past 30-days was reported by 37.3% (*n* = 22) of participants, or 50% of those who reported having smoked 100 cigarettes in their lifetime. Cigarette smoking was more likely in the group with past 30-day cannabis use than the group without (*p* <.001). ENDS use was reported by 35.6% of participants but was not different between groups based on cannabis use (*p* = .076). Combined, 59.3% of the sample used some form of tobacco or nicotine consumption in the past 30-days (cigarettes or vaping). Notably, 19.4% of participants who reported past 30-day cannabis use reported no use of tobacco.

#### Quit attempts

Most participants with past 30-day cannabis use (total *n* = 36) reported that they had no desire to quit (*n* = 26, 72.2%). Only three participants (8.3%) with past 30-day cannabis use reported a current desire to quit, with five reporting they had already quit within the past 30-days (13.9%). Motivation to quit was low in this group, (mean = 3.25, SD = 3.30, range: 0-7), with all three participants who indicated a desire to quit reporting that pregnancy had *increased* their motivation to use cannabis. Despite this, multiple cannabis quit attempts were reported by 50% of participants with past 30-day use (*n* = 18). Of those, 27.8% had tried to quit once, 27.8% twice, 22.2% tried to quit 3 times, 16.7% tried to quit 5 times, and one respondent attempted to quit cannabis use 13 times (5.6%). In the full sample, quit attempts were reported by 58.9% (*n =* 33), with 54.8% of those having attempted to quit on more than one occasion (range: 2-13). The most common quit method was "cold turkey." No one reported trying inpatient or outpatient therapy, such as counseling to aid in cannabis cessation. There were no differences in quit attempts between those who endorsed using cannabis in the past 30 days compared to those who did not (*p* = .425; **Table 3**).

**Table 3:** Quit attempts and reasons for abstinence from cannabis and tobacco (full sample *n* = 59).

Questions:	Full sample *n* (%) mean [SD]	No past 30-day cannabis *n =* 23 (39.0%)	Past 30-day cannabis *n* = 36 (61.0%)	*p*
Cannabis				
Ever Attempted to Quit	33 (58.9)	15 (65.2)	18 (50.0)	.302
Past Quit Attempts (range: 1-13)	2.42 [2.39]	1.54 [.97]	3.06 [2.84]	.118
Among Those with Past 30-Day Cannabis Use (*n* = 36)				
Reasons to Abstain				
Concern about effects on Baby/pregnancy	20 (33.9)		20 (87.0)	
Uncertain about safety	5 (8.5)		5 (21.7)	
Just didn't want to	4 (6.8)		2 (8.7)	
Illegal where I live	2 (3.4)		2 (3.4)	
Other	2 (3.4)		3 (13.0)	
Want to Quit Cannabis Now	3 (5)			
Among Those with Past 30-Day Tobacco Use	*n* = 35	*n =* 6	*n =* 29	
Reasons to Abstain from Tobacco				
Concern about effects on baby/pregnancy	5 (14.3)	0 (0)	5 (17.2)	.366
Uncertain about safety	0 (0)	–	–	–
Cost	1 (2.9)	1 (1.7)	0 (0)	.171
Just didn't want to	4 (11.4)	0 (0)	4 (13.8)	.454
Other	5 (14.3)	2 (33.3)	3 (10.3)	.195
Ever Attempted to Quit Tobacco	29 (82.9)	14 (60.9)	28 (77.8)	.303
Past Quit Tobacco Attempts (range 0-10)	2.62 [2.06]	1.60 [1.34]	3.36 [3.15]	.307
Want to Quit Tobacco Now	15 (42.9)	1	4	.714
Motivation to Quit Tobacco (range 3-10)	5.80 [2.24]	6.15 [2.19]	3.50 [.71]	.077

No participants endorsed cost or unavailability for purchase as reasons to abstain from cannabis.

Nearly half (*n* = 15, 44.1%) of participants with past 30-day tobacco (total *n* = 35) use reported a current desire to quit, with two participants (14.3%) reporting that they were able to quit in the past 30-days. Past tobacco quit attempts were reported by more than half of participants (54%) ranging from 1 and 14 attempts. The most frequently reported method used to attempt to quit in the past was "cold turkey" at 56%. Nicotine replacement therapy in the form of gum or patches was used by 17%. A few respondents reported attempting to wean or taper off cigarettes unsuccessfully (1.7%), using e-cigarettes or vaping (1.7%), or using phone games (1.7%) as a method of cessation. No one reported using behavioral counseling to aid in tobacco cessation. Most respondents with current use of tobacco or nicotine (75%) reported some motivation to quit using tobacco (mean = 5.8, SD = 2.24, ranging from 3–10 where 0 is not prepared to quit and 10 is already quit). There were not differences in tobacco quit attempts between those with or without past 30-day cannabis use (*p* = .368).

### Substance use motivations for use and reasons for abstinence

Most participants endorsed multiple motivations for use (mean = 5.49, SD = 4.08). Five or more motivations were endorsed by 42.4% of the sample. Frequencies of endorsing each motivation are reported for the full sample and by current cannabis use groups (past 30-day use and no past 30-day use) in **Table 4**.

**Table 4:** Motivations for cannabis use were elicited using a panel of 18 possible responses to the prompt: "Select all the reasons you have used cannabis during this pregnancy".

Questions:	Full sample *n* (%)	No past 30-day use *n* = 23 (39.0%)	Past 30-day use *n =* 36 (61.0%)	p	OR (95% CI)
Psychological symptoms					
To manage anger	18 (30.5)	1 (4.3)	17 (47.2)	<.001	19.68 (2.39-162.06)
Helps with emotional pain	28 (47.5)	4 (17.4)	24 (66.7)	<.001	9.50 (2.64-34.23)
To reduce fear/anxiety	29 (49.2)	5 (21.7)	24 (66.7)	<.001	7.20 (2.15-24.12)
To reduce stress	36 (61.0)	8 (34.8)	24 (66.7)	.017	3.75 (1.25-11.30)
To relax	29 (49.2)	10 (43.5)	19 (52.8)	.486	
Physical symptoms					
Helps with nausea or morning sickness	44 (74.6)	11 (47.8)	33 (91.7)	<.001	12.00 (2.85-50.52)
Helps with sleeping	36 (61.0)	7 (30.4)	29 (80.6)	<.001	9.47 (2.82-31.83)
Helps with physical pain	33 (55.9)	8 (34.3)	25 (69.4)	.009	4.26 (1.40-12.97)
Helps with other medical conditions	14 (23.7)	3 (13.0)	11 (30.6)	.123	
Lifestyle/enjoyment					
Increases the enjoyment of other activities (TV, music, etc.)	16 (27.1)	2 (8.7)	14 (38.9)	.011	6.68 (1.35-33.02)
To help me avoid using other substances (tobacco, alcohol, drugs)	12 (20.3)	3 (13.0)	9 (25.0)	.266	
I like the feeling (being high)	10 (16.9)	3 (13.0)	7 (19.4)	.523	
I feel more spiritual and/or self-reflective	8 (13.6)	1 (4.3)	7 (19.4)	.099	
To enhance creativity	7 (11.9)	1 (4.3)	6 (16.7)	.154	
Helps me socialize with others	7 (28.8)	1 (4.3)	6 (16.7)	.154	
People who are important to me use it	1 (1.7)	0	1 (2.8)	.420	
Improves the effects of alcohol and/or other drugs	0	0	0		
Saw information or stories promoting it	0	0	0		

Frequencies and percentages are reported for those who endorsed the response within each group. Odds ratios (OR) and 95% confidence intervals (CI) are reported for motivations with *p* <.05.

#### Motivations related to psychological symptoms

Individuals who reported using cannabis to manage anger were 19.68 times more likely to report past 30-day cannabis use than those who did not endorse this motivation (OR = 19.68, 95% CI 2.39-162.06, p <.001), with nearly half of those with past 30-day use reporting this motivation compared to only 1 person without. Other psychological motivations were reported more frequently, with 'helps with emotional pain' (OR = 9.50, 95% CI 2.64-34.23), 'to reduce fear/anxiety' (OR = 7.20, 95% CI 2.15-24.12), and 'to reduce stress' (OR = 3.75, 95% CI 1.25-11.29, *p* = .017) significantly predicting past 30-day use.

#### Motivations related to medical use or physical symptoms

Nausea or morning sickness was the most commonly reported motivation among the physical symptoms and significantly predicted past 30-day use (OR = 12.00, 95% CI 2.85-50.52, p <.001). Using cannabis to help with sleep was 9.47 times more likely to occur in the past 30-day cannabis use group (OR = 9.47, 95% CI 2.82-31.83, *p* <.001). Similarly, those who reported using cannabis because it helps with physical pain were 4.26 times more likely to report past 30-day cannabis use than those who did not endorse this motivation (OR = 4.26, 95% CI 1.4-12.97, *p* = .009).

#### Motivations related to lifestyle or enjoyment

Endorsing "increases the enjoyment of other activities" was associated with a 6.68 increase in likelihood of past 30-day cannabis use compared to those who did not endorse this motivation (OR = 6.68, 95% CI 1.35-33.02, *p* = .011). Enjoyment of other activities was the only motivation in this category that predicted differences between past 30-day use and non-use; however other motivations were commonly endorsed including 'to help me avoid other substances (tobacco, alcohol, drugs)' and enjoyment of the feeling of being high. No individuals endorsed 'improves the effects of alcohol and/or other drugs' or 'saw information or stories promoting it' (**Table 4**).

#### Multiple motivations for cannabis use

To determine the usefulness of composite motivations in the model, we initially fit a logistic regression using only maternal age and current smoking status (past 30-day use of cigarettes) as predictors. The model omnibus logistic regression test was significant (*χ^2^* = 13.51, *df* = 2, *p* = .001) with a Cox & Snell *R^2^* of 0.26. Current smoking was predictive of current cannabis use (*β* = 2.58, SE = .88, *p* = .003) but maternal age was not (*p* = .546). Odds of endorsing current cannabis use were 13.13 times higher among those with current cigarette smoking compared to those who did not endorse recent cigarette use before accounting for motivations.

In the full model, including the motivations indicator (composite of 18 motivations) improved the model fit and proportion of variance explained (Cox & Snell *R^2^* = .36). The logistic regression omnibus test was again significant (*χ^2^* = 19.64, *df* = 3, *p <*.001). Current smoking remained a significant predictor, though attenuated (*β* = 2.19, SE = .922, *p* = .018), and maternal age remained uninformative (*p* = .155). Endorsing 6 or more motivations was associated with an increase in likelihood of endorsing current cannabis use (*β* = 2.33, SE = 1.05, *p* = .027). Odds of endorsing current cannabis use were 10.28 times more likely for those who endorsed 5 or more motivations compared to those who endorsed fewer, and 8.91 times more likely for current cigarette smokers compared to nonsmokers. Results of logistic regression models are presented in **Table 5**.

**Table 5:** Prediction of past 30-day cannabis use via number of motivations endorsed.

Model	*b*	SE-b	Wald	*df*	*p*	Exp (B) OR	95% CI	Cox & Snell R^2^
Model 1 (empty)			13.51	2	.001			.264
Intercept	-1.53	2.53	0.37		.546	0.217		
Maternal age	.05	.10	0.28		.641	1.05	0.86-1.27	
Current smoker	2.58	0.88	8.65		.003	13.13	2.36-73.07	
Model 2 (full)			19.64	3	<.001			.360
Intercept	-8.93	4.81	3.40		.065	0		
Maternal age	0.20	0.14	2.03		.155	1.225	0.93-1.62	
Current smoker	2.19	0.92	5.63		.018	8.91	1.46-54.32	
Motivations (5+)	2.33	1.05	4.90		.027	10.29	1.31-81.13	

Binary logistic regression was used with past 30-day cannabis use (yes/no) as the dependent variable.

A *post hoc* sensitivity test to examine whether effects were driven by heavier use patterns in early pregnancy (e.g., potentially before participants knew they were pregnant) was also conducted using binary logistic regression with the same predictors as the full model. For this analysis, the sample was limited to participants in the second and third trimester only (*n* = 34). The model remained statistically significant in the smaller sample (*p* = .019) with no change in explained variance (Cox & Snell *R^2^* = .36).

#### Reasons for abstinence from cannabis and tobacco use

The most common reason selected to avoid cannabis use was "concerns about effects on baby and pregnancy." All 20 participants who endorsed this reason abstained from cannabis use in the past 30 days, and only 3 individuals who did not endorse this reason abstained (*p* <.001). Concerns about safety also showed a significant relationship to past 30-day use, with all five participants who endorsed this reason abstaining (*p* = .003). All other reasons for abstaining received fewer than 5 endorsements, and none were statistically significant as predictors of abstinence (*p*s >.05). Participants were asked about reasons for abstinence from tobacco only if they reported smoking or vaping tobacco or nicotine in the past 30 days (*n* = 35). They endorsed the following reasons for abstinence from tobacco: concerns about effects on baby/pregnancy (*n* = 5, 14.3%), cost (*n* = 1, 2.9%), 'just didn't want to' (*n* = 4, 11.4%), and some other reason (*n* = 5, 14.3%) including two participants who abstained from smoking due to vaping instead. No participants endorsed 'uncertain about safety' of tobacco and no reasons for abstinence of tobacco were significant predictors of past 30-day cannabis use (**Table 3**).

## Discussion

The present study examined cannabis use patterns, motivations for use, co-use with tobacco and nicotine, reasons for abstinence, and quit attempts in a sample of pregnant persons who self-reported cannabis use during pregnancy. Motivations to use cannabis predicted past 30-day use of cannabis, both individually and as a composite measure. These findings are aligned with the behavioral science theories that underpin research into motivations of risk behaviors, including the Health Beliefs Model and Theory of Planned Behavior. These theoretical frameworks posit that behavior is driven by motivations and influenced by perceptions of safety and harm (24, 35). These and other theories of behavior and behavior change highlight the importance of understanding motivations for substance use behaviors. Our data support this model, with motivations for use significantly predicting behavior, while participants reported concerns about effects on baby/pregnancy and uncertainty about safety as common reasons for abstinence from the behavior.

Perhaps most concerning, our findings demonstrate that pregnant women who continue cannabis use may be using cannabis at particularly high frequency. More than half our sample endorsed daily use and more than 4 in 5 used cannabis on at least 15 days of the past 30. Cannabis has a dose-dependent relationship with low birth weight (5, 6), a factor that provides biological plausibility for similar dose-dependent relationships with other adverse outcomes. Given the 5-fold increase in THC potency documented over the last two decades (4), this heavy use indicates an urgent need to examine outcomes of prenatal exposures, including for neonates, children, and pregnant persons.

Nearly all participants smoked cannabis, with many also using edibles and vapes. This aligns with broader trends in high-potency product availability following legalization efforts (36, 37). Smoking and vaping pose inhalation risks and may reflect preferences for faster symptom relief (36, 38). These modes also suggest increased cumulative THC exposure, which could heighten fetal risk and dependence potential (39). Co-use of tobacco was common and strongly associated with continued cannabis use. Over 88% of participants had a history of tobacco or nicotine use, and over one-third smoked cigarettes in the past 30 days. This overlap reinforces prior evidence that cannabis and tobacco use often cluster and may compound risks during pregnancy (40). Integrated screening and cessation approaches are needed to address both behaviors simultaneously.

Historic research in prenatal substance use has centered on neonatal outcomes with minimal examination of outcomes specific to the pregnant person. However, recent evidence suggests that heavy cannabis use (> 100 times in the life span) may contribute to excess all-cause and cardiovascular mortality in women (but not men) compared to those who never used cannabis (41); this increase remains after adjusting for age, socioeconomic status, smoking history, alcohol, a range of comorbidities, and antidepressant use. In short, women may be particularly susceptible to cardiovascular complications up to and including mortality related to cannabis use. Pregnancy-related cardiovascular complications are a key driver in the maternal mortality crisis unfolding in the U.S., yet little is known about the outcomes of prenatal cannabis use on pregnant persons (42). Urgent investigation is needed to determine if maternal mortality is linked to prenatal or preconceptual use of cannabis. Our study revealed that many who continued to use cannabis in pregnancy had previously attempted to quit. The inability to sustain abstinence despite wanting to do so raises suspicion of CUD. Pregnant persons have been the focus of evidence-based public health campaigns that emphasize the dangers of cannabis use in pregnancy, but these often focus on advising abstinence rather than providing resources to achieve it. The recognition and management of cannabis withdrawal symptoms (e.g., anxiety, irritability, sleep disruption, depression, loss of appetite, headache) may be necessary when counseling individuals to abstain who have a history of heavy use (43). Increasing provider knowledge and training on cannabis-related maternal and fetal health concerns is crucial for assuring and supporting educated decision-making and efficient prenatal care (44).

Participants indicated the use of cannabis to treat multiple symptoms, both pre-existing and related to pregnancy, including anxiety, sleep, nausea and vomiting. Such symptoms are common occurrences in pregnancy regardless of substance use. However, in individuals with heavy cannabis use, cannabis has the potential to induce and sustain these problems, suggesting that there may be a potential self-reinforcement of use (19). Regardless of etiology, identifying and treating distressing symptoms with safe and effective interventions may decrease motivations for cannabis use. All pregnant persons should be screened for these symptoms, and clinicians should maintain cannabis-related anxiety, sleep disruption, and vomiting as differential diagnoses in pregnancy. More research on cannabis-induced symptoms in pregnancy (e.g., frequency, character, distinguishment from pregnancy-induced symptoms) is needed.

### Limitations

Despite this study's significant and unique findings, its limitations must be considered. The current study used a convenience sampling strategy by recruiting from prenatal clinics affiliated with an academic medical center in a community with limited racial and ethnic diversity. Thus, generalizability may be limited. Replication in larger and more diverse samples is needed. While the study had minimal missingness on questions of interest to this analysis, a small effect of non-response (4 total missing responses across all analyses) may have been present. While efforts were made to incorporate surrounding communities including rural and Appalachian individuals, the results are prone to selection bias. This is compounded by the need for participants to self-disclose cannabis use to be screened for the study. Some eligible persons may not have been willing to disclose use. Self-report measures also create a risk of social desirability bias. Measures to counter this were the provision of a survey link to take outside the prenatal clinic, assurances of confidentiality, and the use of non-stigmatizing language throughout the study. Participants were informed about each substance using recognizable language (e.g., vaping, weed, gummies, wax) that was tested and adapted through conversations with members of the Survivors Union of the Bluegrass to check researcher understanding with members of the local community. The descriptive statistics reported in this paper were not adjusted for multiple testing due to the exploratory nature of the research, though our primary analysis used a family-wise testing strategy; thus, the possibility of some findings in the descriptive data being by chance alone cannot be ruled out. There were no validated measures of motivation at the time of this study. The development of validated measures for motivations for cannabis use and reasons for abstinence in general populations and in pregnancy are urgently needed. Despite these limitations, the present study offers significant insight into the complex reasons for prenatal cannabis use.

### Clinical implications

The findings related to quit attempts suggest that, particularly among those who used cannabis heavily, quitting may be challenging. Unfortunately, minimal resources exist for pregnant individuals who are struggling to quit use of cannabis. Those who abstained from use in this sample largely reported doing so because of concerns about safety and effects on the baby/pregnancy, consistent with behavior change theories. National health organizations have issued recommendations to educate pregnant persons on the risks of cannabis in pregnancy. The AAP published its first formal guidelines in 2018, warning pregnant and lactating women not to use cannabis (11). Due to potential hazards to fetal health, the Centers for Disease Control and Prevention (CDC) advises against cannabis use during pregnancy (45). Similarly, the ACOG discourages cannabis use during pregnancy, citing insufficient data to prove its safety (12).

Despite these recommendations, clinicians report not following guidelines, citing uncertainty as to how to address positive screening results, as well as lack of knowledge about cannabis use risk to pregnancies and fetuses (46). Education for clinicians who work with perinatal patients is needed, including up-to-date information about the scientific evidence of adverse effects and how to intervene. Additionally, clinicians must be taught to recognize their implicit biases when caring for people who use drugs, particularly in pregnancy where stigma related to usage remains a major reason for patients failing to disclose use (47, 48). Decreasing stigma has the potential to reduce medical mistrust among pregnant persons who use cannabis so that they can disclose use and receive cessation care during their maternal care visits (48).

Healthcare providers are key players in addressing cannabis use by fostering trust through neutral, nonjudgmental communication and shared decision-making (49). Patients value discussions about their experiences and risks of cannabis use when delivered with factual, scientific information (49, 50). However, communication practices and knowledge gaps hinder healthcare providers from discussing cannabis with their patients. At present, we are unable to identify widely available clinician training opportunities specific to perinatal cannabis, though offerings on perinatal substance use more generally may provide some information on cannabis. Over half of providers report being unprepared or hesitant to answer patient questions about safe use (46, 51). This limited understanding of the risks and patient rationales for use of cannabis may lead to avoidance and miscommunication. Provider knowledge about cannabis indications, formulations, interactions, and side effects remains inadequate to meet patient needs (46, 52). Doulas are underutilized but can provide valuable information, education, and emotional support to women on topics such as risk factors and warning signs that may require attention, as well as healthy choices to enhance maternal and infant outcomes (53). Nurses and childbirth educators can be a great resource with additional education and training. Further research and translation of evidence into provider education are essential to improve communication about cannabis use with pregnant patients.

Tailored cessation programs should address diverse patient needs, integrate into maternity care, and be covered by insurance (54). Logistically feasible options, such as in-home or telehealth-based support, have shown promise in other populations (54–56). Continued collaboration between medical and mental healthcare providers is also necessary, as addressing underlying psychological and social factors has been shown to improve cessation outcomes (11).

### Policy implications

Though cannabis use during pregnancy is driven by a variety of motivations such as managing nausea, anxiety, and pain, and is perceived as safer than prescription or over-the-counter medications due to its natural origin, tailored health policies are essential to protect maternal and fetal health (50, 56, 57). Culturally specific public health campaigns should prioritize evidence-based alternatives over fear-based messaging, consistent with recommendations from the AWHONN (54). Messaging strategies must include accessible resources, such as conversational toolkits, written handouts, and electronic materials, to ensure consistency, reliability, and effective communication of risks (50, 54, 57, 58).

Screening for cannabis use during prenatal care is also important but must be non-punitive to encourage disclosure and maintain patient-provider trust. Punitive approaches, such as Child Protective Services referrals, create barriers to care and exacerbate disparities (54, 57). Patients prefer cannabis-specific sections in screening tools, as many do not view cannabis as a drug (50). Screening should focus on connecting individuals to tailored resources rather than penalizing them (57). Guidelines from the ACOG recommend universal screening during initial prenatal visits, paired with education on risks and cessation options (11, 58).

Regulatory strategies, including pregnancy-specific warnings on cannabis labels and mandatory retailer education, can reduce prenatal exposure by increasing awareness and informed decision-making (11, 57). Further research on motivations, intervention effectiveness, and care barriers is needed. Aligning with ACOG and AWHONN recommendations will ensure equitable, evidence-based care and improve maternal and child health outcomes.

Recreational cannabis legalization is expanding rapidly across the U.S., increasing access and potentially influencing public perceptions of safety, including during pregnancy (59, 60). In states with legalized recreational markets, cannabis is often marketed with wellness-oriented language and widely available in high-potency forms, which may reinforce its use for symptom relief during pregnancy (36, 61). These shifts heighten the urgency of proactive public health strategies, including standardized labeling and evidence-based patient education that is publicly available to mitigate risk and prevent misinformation.

Recent policy shifts also reinforce the need for expanded research. In 2025, Kentucky legalized cannabis for medical purposes only (62), marking a significant shift in the state's approach to cannabis regulation. This policy change underscores the critical need to scale and replicate studies such as ours to enable better understanding of how evolving legal landscapes influence pregnant individuals' motivations for cannabis use and their patterns of use during pregnancy. Furthermore, expanding this line of research to include states with both restrictive and permissive cannabis laws will provide valuable insights into the broader public health implications of cannabis legalization and its impact on maternal and fetal outcomes.

The present study examined cannabis use patterns, motivations for use, co-use with tobacco and nicotine, and quit attempts in a sample of pregnant persons who self-reported cannabis use during pregnancy. The data reveal that motivations for use and reasons for abstinence of cannabis is complex, with many participants indicating unsuccessful attempts to quit. More research is urgently needed on prenatal cannabis use in larger, more diverse samples as well as among participants originating from regions where cannabis is legalized to provide a more comprehensive picture of the state of perinatal cannabis use in the United States.

## Data Availability

The raw data supporting the conclusions of this article will be made available by the authors, without undue reservation.
